# Effects of 24-Week Exergame Intervention on the Gray Matter Volume of Different Brain Structures in Women with Fibromyalgia: A Single-Blind, Randomized Controlled Trial

**DOI:** 10.3390/jcm9082436

**Published:** 2020-07-30

**Authors:** Juan Luis Leon-Llamas, Santos Villafaina, Alvaro Murillo-Garcia, Francisco Javier Dominguez-Muñoz, Narcis Gusi

**Affiliations:** Physical Activity and Quality of Life Research Group (AFYCAV), Faculty of Sport Science, University of Extremadura, 10003 Cáceres, Spain; leonllamas@unex.es (J.L.L.-L.); svillafaina@unex.es (S.V.); fjdominguez@unex.es (F.J.D.-M.); ngusi@unex.es (N.G.)

**Keywords:** MRI, physical exercise, pain, virtual reality

## Abstract

Background: Exergame-induced changes in the volume of brain gray matter have not been studied in fibromyalgia (FM). This study evaluates the effects of a 24-week exergame-based intervention on the gray matter volume of different brain structures in patients with FM through magnetic resonance imaging (MRI). Methods: A total of 25 FM patients completed 24 weeks of intervention program, and another 25 FM patients did not receive any intervention. T1-weighted MRI was used to assess brain volume, and FreeSurfer software was used to segment the brain regions. Results: No significant effects on gray matter volume of different structures and total gray matter were found. Conclusions: FM patients did not show significant changes in gray matter brain volume between the control and experimental groups after 24 weeks. FM patients showed significant relationships between peak oxygen consumption (pVO2) and the left and right regions of the hippocampus and the left and right regions of the amygdala.

## 1. Introduction

Fibromyalgia (FM) is a chronic disease characterized by widespread pain associated with other symptoms such as sleep disorders, fatigue, anxiety, depression, stiffness, poor physical fitness, or cognitive dysfunction [1], among others. These symptoms lead to a reduction in the health-related quality of life [2], mainly in women between 40 and 59 years old [3].

Previous studies in FM have shown alterations in metabolic activity, functional connectivity, and regions involved in processing pain (i.e., insula, thalamus, amygdala, hippocampus, among others), known as the “pain matrix” [4], and brain structures [5,6,7,8,9,10,11]. Moreover, patients with FM have shown lower thresholds and higher pain ratios, as well as changes in brain activity [12,13] and brain morphology in gray matter [13]. For example, a study found that FM patients have an accelerated brain gray matter loss, and it was related to the duration of the disease [8]. The authors of another study concluded that there is evidence that volumetric changes of gray matter in the frontal-cingulate cortex and the amygdala might reflect both neurobiological preconditions for central sensitization in FM and plastic changes as consequences of chronic pain input [14].

Pharmacological and nonpharmacological therapies have been used in the management of FM symptoms [15]. In this regard, physical exercise is the one that has accumulated the highest level of evidence to date in reducing the symptoms of this disease. In addition, it has been considered a cheap and effective tool for enhancing pain relief, physical function, and well-being [16,17,18]. It is important to emphasize that exercise has a fundamental role in avoiding the physiological effects of aging, as well as promoting the increase of neurogenesis, angiogenesis, and synaptogenesis [19]. These effects are mainly induced by the brain-derived neurotrophic factor (BDNF), growth and differentiation factor 11 (GDF11), and the vascular endothelial growth factor (VEGF), which directly affect brain plasticity [20].

To our knowledge, no studies have evaluated the exercise-induced changes in brain volume in people with FM. Nevertheless, previous longitudinal studies on exercise-based interventions [21], as well as in other populations such as healthy subjects [22], cognitive impairment [23,24], older adults [24,25], multiple sclerosis [26], or schizophrenia [24,27], have shown volume increases in the hippocampus.

Virtual reality-based exercise (VRE), also known as exergames, has shown benefits in the different populations [28,29]. In patients with FM, this type of intervention has been previously used in a nonimmersive version, obtaining improvements in the overall quality of life, pain, disease, mobility, balance, and fear of falling [30,31]. Furthermore, the effects of exergame-based interventions on the brain dynamics of patients with fibromyalgia have previously been studied, finding changes in brain dynamics that could be related to increased cerebral blood flow [32]. On the other hand, two studies have assessed changes in brain structure after exergame training in older adults [33,34]. Therefore, the effects of exergames on the brain are of great interest to the field of neuroscience. However, the effects of exergame-based interventions on brain volume in patients with FM have not previously been studied. Thereby, this study aims (1) to evaluate the effects of a 24-week exergame-based intervention on the gray matter volume of different brain structures of women with FM by magnetic resonance imaging (MRI) analysis, and (2) to analyze the relationship between aerobic and cognitive capacities with the gray matter volume brain structures. As exercise-based interventions can produce changes in brain morphometry, it is hypothesized that participants would show increased gray matter volumes in the different brain structures after the exergame intervention.

## 2. Materials and Methods

### 2.1. Trial Design

This study was a single-blinded randomized controlled trial. Participants were randomly assigned numbers by one of the researchers who did not participate in the statistical analysis or data acquisition and they were randomly allocated into two groups: the control group (CG) and the exercise group (EG). All evaluations were performed by another researcher who was blinded to the grouping allocation. Participants were not blinded since they were informed of the procedures and knew whether or not they were performing the exercise intervention. All procedures were approved by the University Research Ethics Committee (62/2017). The trial was registered at the International Standard Randomized Controlled Trial Number Registry (ISRCTN65034180), and the protocol is available in BioMed Central website [35]. Participants gave their written consent for the procedures in the study.

Different articles focused on the primary outcomes (i.e., electroencephalography, physical fitness, and quality of life) of the trial have been recently published [32,36,37,38]. Nevertheless, the hypothesis in the present study is entirely novel (improvements in different volumetric brain structures after an exergame intervention) and significantly differs from the other articles. This enables us to deeply examine the findings in the brain volume of women with FM. Furthermore, the scope, audience, and research professionals that this article involves are different and complementary.

### 2.2. Participants

A total of 56 women from a local FM association were recruited for this study and fulfilled the following inclusion criteria: (1) female between 30 and 75 years old; (2) diagnosed with FM by a rheumatologist according to the 2010 criteria established by the American College of Rheumatology; (3) able to communicate with research staff; (4) have read and signed the written informed consent conforming to the updated Declaration of Helsinki. Moreover, participants were excluded if (1) they were pregnant, (2) they had any cerebral injury, (3) illegible MRI sequences were obtained, (4) they had contraindications for physical exercise, or (5) they had changed their usual care therapies during the intervention program. The intervention was carried out in the University facilities (Faculty of Sport Science, Caceres, Spain) from January 2018 to June 2018.

### 2.3. Interventions

The EG participants completed a 24-week training program, whereas the CG participants continued their daily routine. The intervention consisted of two sessions per week (60 min per session). All sessions were conducted in groups of two or three participants in the university facilities, and there were no important adverse events as a result of the intervention. A specialized physical therapist, who conducted participant evaluations, also supervised all the sessions.

The exercise intervention was based on an exergame called VirtualEx-FM that was created by the research group, which aims to improve the ability to develop activities of daily living as well as physical conditioning in FM patients. VirtualEx-FM meets the key points of VR rehabilitation therapy [39] and has been used previously [30,31]. The program focuses on balance, postural control, coordination, mobility, aerobic conditioning, and strengthening of the upper and lower body, providing visual feedback and maintaining the correct execution of movements [31].

A typical session contained the following parts: (1) a video warm-up, where an expert performs joint movements and participants imitate these movements. The speed could be controlled by the expert at 0.5×, 1×, 1.5×, and 2×. (2) An aerobic component through dance steps (Zumba) performed by a dance teacher. (3) A game in which participants have to reach an apple with an avatar, getting to work on postural control and coordination, which was controlled and modified by the physical therapist. (4) Walking training, where participants must complete a virtual trail of footprints on a virtual floor. The interface allows the selection of different types of steps (i.e., normal, heel walking, tiptoe, raised knees, and raised heels; see Collado-Mateo et al. [31] for further details).

### 2.4. Data Collection and Outcomes

All the tests were carried out in the laboratory of the research group. Thus, tests were implemented at the beginning and the end of the intervention program by the same researchers. The variables measured were anthropometric measures, disease impact, cognitive decline, aerobic domain, and gray matter brain volumes.

First, anthropometric measurements of the participants were taken to report the body mass index (BMI). Subsequently, participants completed the Spanish version of the Fibromyalgia Impact Questionnaire (FIQ), which evaluates the impact of symptoms of the disease from 0 to 100, indicating the minimum to maximum impacts, respectively. FIQ is an extensively validated fibromyalgia-specific tool that captures the overall effects of fibromyalgia symptomatology (i.e., pain, fatigue, rested, stiffness, anxiety, depression, physical impairment, feeling good, or work missed) [40,41,42]. After that, trained research staff administered the Mini-Mental State Examination (MMSE), previously used in patients with fibromyalgia [43,44]. MMSE is a widely used test of cognitive function; it includes tests of orientation, attention, memory, language, and visual–spatial skills. A higher score represents a better cognitive state.

The 6-min walk test is a reliable measure in people with fibromyalgia [45]. The results of the 6-min walk test were used to predict peak oxygen consumption (pVO2) [46] through an equation from the distance covered in 6 min [47]. The regression equation to predict pVO2 from 6-min walk distance and BMI is pVO2 (ml/Kg/min) = 21.48 + (−0.4316 × BMI) + (0.0304 × distance (m)).

### 2.5. Image Acquisition

T1-weighted structural MRI scans were acquired from a 3.0 Tesla (T) system (Achieva 3.0T TX, Philips Medical Systems, Best, Netherlands) with an 8-channel receiver head coil. For each T1-weighted structural scan, the parameters were set as follows: 196 slices were acquired; the turbo field echo (TFE) imaging sequence (time repetition/time to echo = 11.51/2.8 ms; matrix size = 256 × 256; flip angle = 10°; slice thickness = 0.9 mm; number of averages = 1) was used.

### 2.6. Image Processing

All T1-weighted images were processed using the FreeSurfer software 6.0 version, a program freely available for download (Laboratory for Computational Neuroimaging, Athinoula A. Martinos Center for Biomedical Imaging, Charlestown, MA, USA) [48]. Automated segmentation of the T1-weighted images was used employing the recon-all command [49] on a MacBook Pro (Version OS X 10.14, 8GB, 2.30 GHz, Intel Core i5).

Preprocessing data include the following steps: (1) motion correction and averaging [50]; (2) removal of nonbrain tissue [51]; (3) automated Talairach transformation [52]; (4) segmentation of subcortical white matter and deep gray matter structures [53]; (5) intensity normalization [54]; (6) tessellation of the gray matter and white matter boundary [55]; (7) topology correction; (8) surface deformation following intensity gradients to reconstruction [53]. Structures that are part of the “pain matrix” (left and right regions of the hippocampus, insula, thalamus, amygdala, and cerebellum, in addition to total gray matter) and have an interest in the study of FM [56,57] were selected.

### 2.7. Statistical Analyses

The SPSS statistical package version 24 (IBM Corp, Armonk, New York, United States) was used to analyze the data.

To conduct the intention-to-treat analysis by multiple imputations (MIs) of missing values, the data from all 55 participants were used following the Sterne et al. guidelines [58]. Our missing data were classified as missing at random. The SPSS software package was used for the MIs of data.

Nonparametric tests were conducted because the dataset was not large and the data were not always Gaussian. To explore the effectiveness of the exergame-based intervention, the Mann–Whitney U-test was conducted to examine the differences between groups for each variable. Moreover, within-group comparisons were conducted by the Wilcoxon signed-rank test.

The Benjamini–Hochberg false discovery rate correction for multiple comparisons was applied in each comparison to avoid Type I errors. The partial eta-squared effect size was reported for each statistical test [59]. According to Cohen [60], effect sizes could be classified as small (0.01 ≤ *η*² < 0.06), medium (0.06 ≤ *η*² < 0.14), and large (*η*² ≥ 0.14).

Furthermore, Spearman’s rho correlation analyses were used to evaluate the relationship between pVO2, MMSE, and brain structure volumes.

## 3. Results

**Figure 1:**
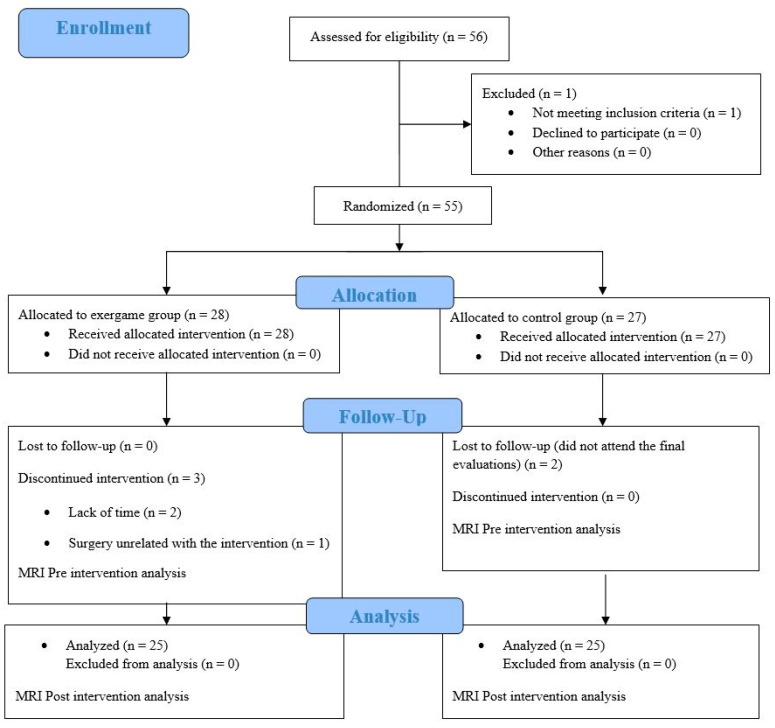
Flow chart of participants.

The flow diagram for participants is represented in Figure 1. A total of 56 women with FM were screened for eligibility. One participant was excluded for not meeting with the inclusion criteria. Therefore, 55 women were randomly allocated into two groups: CG and EG. Two women were not able to attend the final evaluations for CG. On the other hand, three women from EG were excluded by lack of time (*n* = 2) and a surgery unrelated to the exercise intervention (*n* = 1).

Table 1 shows the baseline characteristics of the participants of both groups. Differences between EG and CG were not observed in age, the impact of the disease, years with FM symptoms, peak volume oxygen consumption, MMSE, anthropometric characteristics, and gray matter brain volumes (*p*-value > 0.05).

Table 2 and Table 3 show the efficacy and the intent-to-treat analyses, respectively. Significant differences in the effect of the exergame-based intervention were not found for the gray matter volumes of the different brain structures, the total volume of gray matter, the pVO2, and the MMSE score.

Table 4 shows the relationship at baseline between the pVO2, the MMSE, and the gray matter brain volumes. The analyses revealed a relationship between the pVO2, the left and right regions of the hippocampus, and the left and right regions of the amygdala (*p*-value < 0.05).

## 4. Discussion

This study is the first randomized controlled trial that examines the effects of an exergame-based intervention on the gray matter volume of different brain structures in patients with FM. The intervention did not show any overall effect in the volume of some brain structures involved in the “pain matrix” (hippocampus, amygdala, thalamus, insula, and cerebellum) as well as the total gray matter of the brain in the comparison of CG and EG.

Results showed that no statistically significant effects were found after 24 weeks of exergame intervention in the brain volumes studied. In line with our results, Firth et al. [24] reported in a review that aerobic exercise did not increase the hippocampus gray matter volumes, but produced retention of gray matter in the left hippocampus. However, this information should be taken with caution due to the heterogeneity of the groups and the programs included in this review. Along the same lines, a recent exergame-based study did not show increases in total gray matter and the hippocampus in older adults [61]. Regarding the thalamus, three studies did not show increases in volume after physical exercise interventions [25,26,62]. However, there are previous studies that have reported significant brain volume changes after exercise interventions. In this regard, Wittfeld et al. [63] found volumetric increases in the thalamus as well as in the cerebellum after an intervention with 2103 adults. Moreover, other previous studies have reported effects on the insula volume after aerobic [64] and dance interventions [65]. Considering the large variability of intensities, volumes, or types of training that have reported volumetric changes after physical exercise interventions, future research should elucidate how these variables (i.e., volume, intensity, and type of training) modulate brain volume changes. In addition, if dance interventions are performed, consideration should be given to whether the exercises performed are creative or repetitive, as they influence neuronal survival and brain volume changes [65].

The duration of the intervention might explain why significant results were not achieved. In this regard, previous studies have reported increases in different brain structures, mainly in the hippocampus, after twelve months of an intervention [25,66,67]. Furthermore, this hypothesis may be reinforced by the results of Nieman et al. [66], where they did not find volumetric changes after six months of intervention but did after 12 months. However, the 24-week exergame-based intervention has previously shown significant effects in physical fitness [36,38], quality of life and pain [36], autonomic modulation [37], and even in the EEG beta power spectrum [32] in women with FM. Interestingly, this could indicate that brain volume-related changes need more time to be achieved. Therefore, future studies should investigate the role of intervention duration in brain volume changes.

However, the within-group analysis showed significant changes in both intervention and control groups. An analysis of different brain volumes in the same group of patients may represent a critical step in elucidating the central mechanisms of FM. The significant changes within groups are in line with previous morphometric reports of changed gray matter volumes in FM patients [68]. Another study has reported volumetric changes to be more pronounced with longer exposure to FM pain. In that case, the authors explained that a longer FM duration reflects a compensatory mechanism in which the brain may attempt to prevent the negative effects of constant nociceptive input [69]. It is also possible that the morphometric changes reflect an increase in the affective–evaluative processing of pain, as has been suggested to occur in patients with chronic lower back pain [70].

The correlations of the present study provide interesting information regarding the significant relationships found between pVO2 and the right and left regions of the hippocampus and the right and left regions of the amygdala. Similar to our results, another study has indicated that higher levels of aerobic fitness are associated with higher hippocampal volumes in older humans [71]. Consequently, it is known that aerobic exercise increases cerebral blood volume in the dentate gyrus of adults, which is related to neurogenesis [72]. On the other hand, the significant relationships found between pVO2 and the right and left regions of the amygdala may be in line with the results of a study that analyzed neural activity and respiratory frequency on anticipation of anxiety. In the most anxious subject, electric current sources were found in the left amygdala. The activation of this area participates in the enhancement of respiratory frequency [73]. Thus, it could be interesting to study the relationship between the aerobic system and the amygdala, also taking into account that the population with FM is a population related to anxiety symptoms [74,75,76].

The present study has some limitations. First, we only included women with FM, so we cannot generalize the results to men with FM. In addition, the women of the present study have different ages, being an aspect to consider in gray matter studies [77]. Second, the effect of the exergame intervention cannot be isolated since no group performed traditional exercise training. Finally, the intensity of training was not specified since the personal conditions of the participants were changing due to the disease. However, this is the first study that evaluates the effects of an exergame tool on different gray matter volumes of brain structures in women with FM.

## 5. Conclusions

The exergame-based intervention did not induce significant changes in gray matter brain volume when comparing CG and EG of women with FM. The patients of the present study showed significant relationships between pVO2 with the left and right regions of the hippocampus and the left and right regions of the amygdala. Future research on the determinants of the sensitivity to exercise-specific brain changes are warranted.

## Figures and Tables

**Table 1:** Descriptive characteristics of participants and differences between groups at the baseline of fibromyalgia patients.

Variable	Exercise Group Median (IQR)	Control Group Median (IQR)	Value of the Contrast	*p*-Value
Sample size	25	25		
Age (Years)	54.00 (16.00)	53.00 (13.00)	−0.351	0.800
Height (cm)	160.00 (11.00)	159.00 (7.00)	−0.351	0.800
Weight (Kg)	69.30 (16.20)	72.35 (19.40)	−0.470	0.800
BMI (Kg/m²)	27.00 (4.30)	28.35 (7.40)	−0.650	0.800
FIQ-100	57.58 (28.47)	63.90 (23.56)	−0.490	0.800
Years with FM	8.50 (10.75)	11.00 (10.25)	−0.308	0.800
pVO2 (ml/Kg/min)	23.77 (4.24)	24.46 (5.38)	−0.019	0.985
MMSE	29.00 (1.00)	28.50 (2.25)	−2.151	0.460
Left Hippocampus	3.04 (0.43)	2.93 (0.36)	−1.660	0.460
Right Hippocampus	3.08 (0.31)	3.07 (0.33)	−0.760	0.800
Left Insula	3.35 (2.06)	3.34 (2.49)	−0.919	0.800
Right Insula	3.82 (1.70)	4.28 (1.21)	−0.809	0.800
Left Amygdala	1.37 (0.19)	1.41 (0.16)	−0.319	0.800
Right Amygdala	1.63 (0.18)	1.64 (0.22)	−0.873	0.800
Left Thalamus	7.83 (0.94)	7.51 (0.61)	−1.532	0.477
Right Thalamus	7.07 (0.81)	6.81 (0.66)	−1.724	0.460
Left Cerebellum	48.37 (9.92)	46.12 (8.09)	−0.958	0.800
Right Cerebellum	51.39 (13.14)	49.04 (11.27)	−1.681	0.460
Total Cerebral GM	436.10 (72.50)	428.35 (126.54)	−0.319	0.800

Abbreviations: IQR, interquartile range; BMI, body mass index; FIQ, fibromyalgia impact questionnaire; FM, fibromyalgia; pVO2, peak volume oxygen consumption; MMSE, mini-mental state examination; GM, gray matter.

**Table 2:** Efficacy analysis of the effects of exergame intervention in patients with fibromyalgia on the different brain structures, the pVO2, and the MMSE.

Variables				Between Group Comparison			Within Group Comparison		
Brain Areas (cm³)	Groups	Pre Median (IQR)	Post Median (IQR)	Value of the Contrast	*p*-Value	Effect Size	Value of the Contrast	*p*-Value	Effect Size
L. Hippocampus	EG	3.04 (0.43)	3.15 (0.22)	−0.342	0.925	−0.020	−2.738	0.016	−0.323
	CG	2.93 (0.36)	3.07 (0.24)				−2.372	0.039	−0.438
R. Hippocampus	EG	3.08 (0.31)	3.26 (0.36)	−0.075	0.925	0.071	−3.011	0.013	−0.354
	CG	3.07 (0.33)	3.15 (0.26)				−2.220	0.048	−0.354
L. Insula	EG	3.35 (2.06)	5.08 (3.16)	0.013	0.925	0.053	−3.320	0.007	−1.037
	CG	3.34 (2.49)	5.93 (1.12)				−2.896	0.016	−1.245
R. Insula	EG	3.82 (1.70)	5.76 (2.41)	−0.450	0.925	0.000	−2.516	0.022	−0.578
	CG	4.28 (1.21)	5.87 (1.23)				−2.451	0.036	−0.751
L. Amygdala	EG	1.37 (0.19)	1.39 (0.17)	−0.501	0.925	-0.090	−0.633	0.527	0.088
	CG	1.41 (0.16)	1.39 (0.11)				−0.087	0.931	0.003
R. Amygdala	EG	1.63 (0.18)	1.68 (0.27)	−0.103	0.925	0.197	−2.776	0.016	−0.329
	CG	1.64 (0.22)	1.65 (0.15)				−1.860	0.091	−0.290
L. Thalamus	EG	7.83 (0.94)	7.72 (0.80)	−0.918	0.925	−0.145	−4.107	0.020	0.272
	CG	7.51 (0.61)	7.41 (0.65)				−4.074	0.075	0.208
R. Thalamus	EG	7.07 (0.81)	6.95 (0.73)	−1.599	0.371	−0.338	−1.834	0.097	0.153
	CG	6.81 (0.66)	6.74 (0.48)				−0.991	0.373	−0.037
L. Cerebellum	EG	48.37 (9.92)	50.80 (8.43)	1.601	0.706	-0.397	−1.705	0.114	−0.158
	CG	46.12 (8.09)	48.27 (5.87)				−2.833	0.016	−0.466
R. Cerebellum	EG	51.39 (13.14)	52.00 (6.91)	−1.483	0.377	-0.489	−1.282	0.236	−0.093
	CG	49.04 (11.27)	50.36 (8.96)				−2.798	0.016	−0.426
Total Cerebral GM	EG	436.10 (72.50)	507.24 (92.04)	−0.595	0.814	-0.177	−3.750	< 0.001	−0.939
	CG	428.35 (126.54)	521.54 (71.29)				−2.972	0.016	−0.965
pVO2 (ml/Kg/min)	EG	23.77 (4.24)	24.51 (3.86)	−1.911	0.371	0.462	−0.807	0.455	−0.108
	CG	23.62 (5.23)	23.02 (5.52)				−1.601	0.142	0.132
MMSE	EG	29.00 (1.00)	29.00 (2.50)	−0.983	0.706	−0.108	−1.996	0.075	0.404
	CG	28.00 (2.00)	28.00 (4.00)				−0.945	0.373	0.248

Abbreviations: IQR, interquartile range; GM, gray matter; pVO2, peak volume oxygen consumption; MMSE, mini-mental state examination; EG, exercise group; CG, control group; L, left; R, right.

**Table 3:** Intent-to-treat analysis of the effects of exergame intervention on the different brain structures, the pVO2, and the MMSE.

Variables				Between Group Comparison			Within Group Comparison		
Brain Areas (cm³)	Groups	Pre Median (IQR)	Post Median (IQR)	Value of the Contrast	*p*-Value	Effect Size	Value of the Contrast	*p*-Value	Effect Size
L. Hippocampus	EG (*N* = 28)	3.10 (0.44)	3.17 (0.27)	−0.511	0.853	−0.057	−2.846	0.011	−0.304
	CG (*N* = 27)	2.95 (0.37)	3.07 (0.23)				−2.560	0.026	−0.434
R. Hippocampus	EG (*N* = 28)	3.11 (0.31)	3.28 (0.36)	−0.435	0.853	0.086	−3.155	0.009	−0.348
	CG (*N* = 27)	3.09 (0.35)	3.16 (0.29)				−2.239	0.058	−0.369
L. Insula	EG (*N* = 28)	3.44 (1.98)	5.20 (2.64)	−0.549	0.853	0.107	−3.628	< 0.001	−1.031
	CG (*N* = 27)	3.57 (2.10)	5.76 (1.24)				−3.628	< 0.001	−1.283
R. Insula	EG (*N* = 28)	4.00 (1.73)	5.71 (2.26)	−0.261	0.853	0.023	−2.951	0.011	−0.611
	CG (*N* = 27)	4.24 (1.66)	5.78 (1.24)				−2.600	0.026	−0.724
L. Amygdala	EG (*N* = 28)	1.37 (0.19)	1.39 (0.17)	−0.301	0.853	−0.150	−0.557	0.593	0.127
	CG (*N* = 27)	1.39 (0.16)	1.39 (0.13)				−0.237	0.816	−0.025
R. Amygdala	EG (*N* = 28)	1.64 (0.19)	1.69 (0.27)	−0.187	0.853	0.155	−2.900	0.011	-0.302
	CG (*N* = 27)	1.63 (0.22)	1.65 (0.17)				−1.989	0.088	−0.309
L. Thalamus	EG (*N* = 28)	7.92 (0.97)	7.73 (0.83)	−0.559	0.853	−0.159	−2.774	0.030	0.288
	CG (*N* = 27)	7.53 (0.63)	7.41 (0.66)				−2.050	0.088	0.222
R. Thalamus	EG (*N* = 28)	7.09 (0.81)	6.95 (0.75)	−1.832	0.355	−0.330	−1.957	0.082	0.161
	CG (*N* = 27)	6.86 (0.68)	6.74 (0.50)				−0.836	0.468	−0.012
L. Cerebellum	EG (*N* = 28)	48.18 (8.60)	50.53 (8.12)	−1.138	0.712	−0.368	−1.878	0.098	–1.169
	CG (*N* = 27)	46.36 (7.46)	49.01 (6.09)				−3.031	0.013	−0.447
R. Cerebellum	EG (*N* = 28)	52.25 (11.40)	52.18 (7.39)	−1.851	0.355	−0.514	−1.087	0.360	−0.066
	CG (*N* = 27)	49.41 (10.03)	50.36 (7.63)				−2.991	0.016	−0.408
Total Cerebral GM	EG (*N* = 28)	438.64 (80.28)	498.36 (88.08)	−0.962	0.787	−0.177	−3.821	< 0.001	−0.852
	CG (*N* = 27)	432.38 (125.40)	520.53 (68.98)				−3.211	0.007	−0.935
pVO2 (ml/Kg/min)	EG (*N* = 28)	23.87 (4.28)	24.62 (3.88)	−2.189	0.355	0.466	−1.139	0.301	−0.126
	CG (*N* = 27)	24.08 (5.19)	23.02 (5.37)				−1.685	0.126	0.134
MMSE	EG (*N* = 28)	29.00 (2.00)	29.00 (2.50)	−1.098	0.712	-0.109	−2.113	0.055	0.388
	CG (*N* = 27)	29.00 (2.00)	28.00 (4.00)				−0.945	0.407	0.231

Abbreviations: IQR, interquartile range; GM, gray matter; pVO2, peak volume oxygen consumption; MMSE, mini-mental state examination; EG, exercise group; CG, control group; L, left; R, right.

**Table 4:** Relationships between the pVO2, the MMSE, and the brain structure volumes.

Variables		pVO2	MMSE
L. Hippocampus	Correlation coefficient	0.349	0.229
	*p*-value	0.017	0.125
R. Hippocampus	Correlation coefficient	0.478	0.205
	*p*-value	0.001	0.173
L. Insula	Correlation coefficient	0.102	0.248
	*p*-value	0.531	0.122
R. Insula	Correlation coefficient	0.102	−0.137
	*p*-value	0.506	0.369
L. Amygdala	Correlation coefficient	0.363	0.144
	*p*-value	0.014	0.346
R. Amygdala	Correlation coefficient	0.360	0.054
	*p*-value	0.015	0.723
L. Thalamus	Correlation coefficient	0.265	0.146
	*p*-value	0.079	0.339
R. Thalamus	Correlation coefficient	0.249	0.259
	*p*-value	0.099	0.085
L. Cerebellum	Correlation coefficient	0.214	0.113
	*p*-value	0.158	0.458
R. Cerebellum	Correlation coefficient	0.288	0.179
	*p*-value	0.055	0.238
Total Cerebral GM	Correlation coefficient	0.194	0.246
	*p*-value	0.201	0.103

Abbreviations: L, left; R, right; pVO2, peak volume oxygen consumption; GM, gray matter; MMSE, mini-mental state examination.
